# Bone transport with magnetic intramedullary nails in long bone defects

**DOI:** 10.1007/s00590-020-02854-5

**Published:** 2020-12-24

**Authors:** Selina Summers, Matija Krkovic

**Affiliations:** 1 School of Clinical Medicine, Addenbrooke’s Hospital, University of Cambridge, Cambridge, CB2 0SP UK; 2 Addenbrookes Major Trauma Unit, Department of Trauma and Orthopaedics, Cambridge University Hospitals, Cambridge, UK

**Keywords:** Bone transport, Bone loss, Femur, Magnetic nail

## Abstract

**Background:**

This study describes the outcomes of internal bone transport with magnetic nails in five cases of traumatic segmental femoral bone defects.

**Methods:**

Five patients with open fracture of the femur and diaphyseal bone loss were included between May 2018 and August 2020. The mean femoral defect was 8.7 cm (range 5.6–16.0).

**Intervention:**

We used plate-assisted bone segment transport (PABST) with PRECICE magnetic nails.

**Results:**

All five patients have fully consolidated. The mean consolidation time and index were 7.5 months and 0.8 mo/cm, respectively. The mean follow-up was 21.3 months. The main complications were reduced knee ROM, mild varus deformity and plate bending. Post-operative SF-36, Oxford Knee scores and ED-5Q-5L scores were also compiled for four of five patients. SF-36 and Oxford Knee scores were reported without pre-injury data for comparison. ED-5Q-5L index and VAS were compared UK population norm and were both found to be statistically insignificant (*p* = 0.071 and *p* = 0.068, respectively).

**Conclusion:**

Bone transport with magnetic nails has the capacity to obtain good functional recovery in long bone defects despite variable outcome pictures. In response to variable outcome reporting in the literature, we propose a standard reporting template for future studies to facilitate more rigorous analyses.

## Introduction

Long bone defects (LBDs) are most commonly caused by high-energy trauma, but also occur as a result of tumour resection and infection. Management of LBDs relies on the principles of distraction osteogenesis pioneered by Ilizarov [1]. The Iliazarov technique forms the foundation in modern reconstructive surgery for limb lengthening and bone transport. However, it involves external fixation which is associated with pin tract infections, systemic infections, soft-tissue tethering and decreased knee range of motion [2, 3]. Recent developments in intramedullary nail lengthening systems, a less invasive approach, reduce the aforementioned device-related risks, nonetheless have issues reported such as mechanical instability and difficulty to control lengthening [4, 5].

Existing intramedullary nail lengthening systems adopt a variety of actuation techniques. Mechanical actuation (e.g. Intramedullary Skeletal Kinetic Distractor and Albizzia) and motorised systems (Fitbone) each  have their specific limitations in distraction control and stability [5].

The recent development of magnetically actuated nails realised advantages over previous implants and a new degree of patient manipulation with an external remote controller (ERC). Such procedure with a fully implantable system requires careful planning to avoid post-operative changes. Konofaos et al. [6] demonstrated an early use of a magnetic bone transport device (M-Bone; Phenix) in patients with primary bone tumours and showed good bone healing. PRECICE nails (NuVasive) are the latest development and have been reported to achieve accurate and precise limb lengthening [7–10]. Plate-assisted bone segment transport (PABST) is principally used in bone transport [11, 12], but modifications of this technique  have been described to improve varus deformity such as by double-plating [13]. The PRECICE nail is controlled using an ERC which, when placed over the nail on the skin, causes magnet rotation which in turn activates a motor that extends or retracts the extendible rod [5].

The treatment technique in LBDs is dictated by the degree of bone loss: either bone transport or acute shortening and re-lengthening, each carrying different risk profiles [14]. While smaller defects (of up to 6–7 cm in femur) are usually managed by acute shortening, the same treatment in larger defects risks soft tissue stacking and neurovascular compromise [14]. Nonetheless, it is a technique accompanied by a known risk of long-term muscle power loss [15]. However, early bone transport was accompanied by serious complications associated with external fixation, and thus, there was a push for development of methods to reduce time in external fixation. Following recent developments, reports have shown a more favourable patient satisfaction rate and complication profile for internal lengthening device over external device [14, 16].

The direction of lengthening is determined by the pattern of bone loss, although some other factors to consider are the age of the patient, size of the intramedullary canal and proximal soft tissue, and the need for deformity correction. Distal bone loss mandates the use of retrograde nails for better control of the final docking position [13]. On the contrary, anterograde nails have the tendency to shift the transport fragment anteriorly towards the docking site if not correctly fixed initially, resulting in missing the docking point.

After the initial treatment, the bone transport surgery is delayed by 6 weeks for any signs of spontaneous bone regeneration [17]. Although there is no clear guideline on the appropriate length of delay, they are not usually less than 3–4 weeks and Wright et al. had allowed for 8–10 weeks to address associated injuries and any active infection [13].

This article describes a series of uses and outcomes of PRECICE magnetic nails in femoral segmental diaphyseal bone defects.

## Patients and methods

Five patients (4 males and 1 female, age 46.8 ± 18.3 years) with open fracture of the femur with BDs were included between May 2018 and August 2020. All patients had traumatic bone loss (mean size 8.7 ± 3.7 cm). One patient is a smoker and one is diabetic. Two patients (Patients 1 and 2) had previously unsuccessful attempts at bone transport by MRS Ex-Fix and pulley system, respectively. Patient 2’s pulley system failure was due to patient non-compliance.

The initial treatment usually consisted of bone debridement and temporary ex-fix. This was followed by AxSOS plate when patient was stable with no sign of infection and fit for surgery. After on average 6 weeks if there was no spontaneous union [17] we proceeded with PRECICE nail and corticotomy. All the operations were performed with the PABST technique and by the same surgeon.

All procedures were performed with the transported segment pulled in a retrograde direction. The distraction process started on the 8th post-operative day at 1 mm/day in 4 steps. Patient 3 moderated the rate to 0.5 mm/day temporarily due to plate bending and risk of breaking. He required several recharging of the nail and exchanged the nail for a shorter one. However, he terminated bone transport by PRECICE nails early due to nail jamming of the nail and switched to MRS ex-fix and transport over nail for final lengthening and docking. The remaining four patients completed bone transport with PRECICE nails.

Three pre-distraction lengths of the PRECICE nail were used: 215, 230 and 245 mm. The former two consist of a telescoping rod that can achieve 50 mm of lengthening, while the last allows for 80 mm of lengthening. AxSOS plates were used in all cases to secure the positions of proximal and distal fragment during the treatment.

Patients were evaluated for consolidation index, time to full weight bearing, time to union, knee range of motion (ROM) and complications. Consolidation time was defined as the end of distraction to full weight bearing in months. Consolidation index was defined as the time from end of bone transport to full weight bearing divided by the transported distance. Time to union was defined as the time from injury to union confirmation at the docking site. Union was assessed by plain radiograph. Knee ROM was assessed in follow-up clinics. After recovery, patients were asked to complete patient-reported outcome measures (PROM) assessments with SF-36 [18], Oxford Knee Score and 5Q-5D-5L [19] surveys. Where applicable, the result was compared to the national average since no pre-operative results were available.

In defect size measurement, we accept an X-ray image magnification of ~ 10% and that it is not equal amongst the patients. The defect size was taken to be the transported distance of the segment. We aimed for 1–2 cm shorter leg than the contralateral leg to enable and improve knee bending and speed up the recovery, and hence ignored limb length discrepancy (LLD).

Patients were allowed to start partial weight bearing as tolerated during transport, but three cases remained non-weight bearing until after transport ended. Full weight bearing was initiated when X-ray radiographs in two dimensions confirmed strong regenerate by blurring the corticotomy lines in either AP or lateral view, unless contraindicated by pain and stiffness. Implants were not removed post-recovery.

## Results

The patient outcomes are summarised in Table 1. Average consolidation index was 0.8 mo/cm (range 0.5–1.3). All progressed to full weight bearing at an average of 18.7 months (range 9.1–30.6). Average follow-up was 21.3 months (range 6.8–33.0), but three patients are still being followed up. Patient 3 had nail failure, while two others had plate bending. Patients 1 and 2 have significantly reduced knee range of movement and are receiving physiotherapy. Patient 5 has yet to confirm union on CT scan.

**Table 1:** Summary of patient characteristics and outcome

Patient	Age/sex	Location	Defect size (cm)	Consolidation time (mo)	Consolidation index (mo/cm)	Time to fwb (mo)	Time to union (mo)
1	30/M	R femur	5.6	7.3	1.3	30.6	30.6
2	40/F	L femur	8.0	6.7	0.8	14.5	12.9
3	29/M	R femur	16.0	15.0	0.9	27.5	21.4
4	77/M	R femur	6.9	4.6	0.7	11.7	7.3
5	58/M	L femur	7.0	3.8	0.5	9.1	NA
Average	46.8		8.7	7.5	0.8	18.7	18.1
Span			5.6–16.0	3.8–15.0	0.5–1.3	9.1–30.6	7.3–30.6

Patient	Knee ROM	F/U (mo)	Complications
1	10–90	33.0	None
2	0–90	25.2	None
3	0–120	26.7	Plate bending, nail failure
4	0–120	15.0	Heterotrophic ossification
5	30–115	6.8	Plate bending, mild varus
Average	8–107	21.3	
Span	0–120	6.8–33.0	

Four out of five patients completed the PROM assessment only after recovery (Table 2). The results for EQ-5D-5L were compared to the UK average. The mean EQ index is 0.536 compared to the national average [20] of 0.856, and the EQ VAS is 51.3 compared to 82.8. The SF-36 and OKS results are presented without a baseline comparison.

**Table 2:** SF36, OKS, EQ-5D-5L survey results. Population norm taken from Janssen and Szende (2014) [20]

PROM	Mean	Median	SD	Range
*SF-36*				
Physical functioning	25	17.5	20.4	10–55
Social functioning	37.5	75	43.3	0–75
Physical limitation of role	0	0	0.0	0–0
Emotional limitation of role	75	100	50.0	0–100
Mental health	61	62	26.6	36–84
Vitality/energy	37.5	35	33.0	5–75
Pain	38.8	38.8	32.0	0–77.5
Global health	46.3	45	22.5	20–75
Health change	43.8	50	31.5	0–75
*OKS (0–48)*	20.3	19	9.6	11–32
*EQ-5D-5L*				
EQ index	0.536	0.607	0.324	0.112–0.816
Population norm	0.856			
EQ VAS	51.3	55	31.2	10–85
Population norm	82.8			

**Fig. 1:**
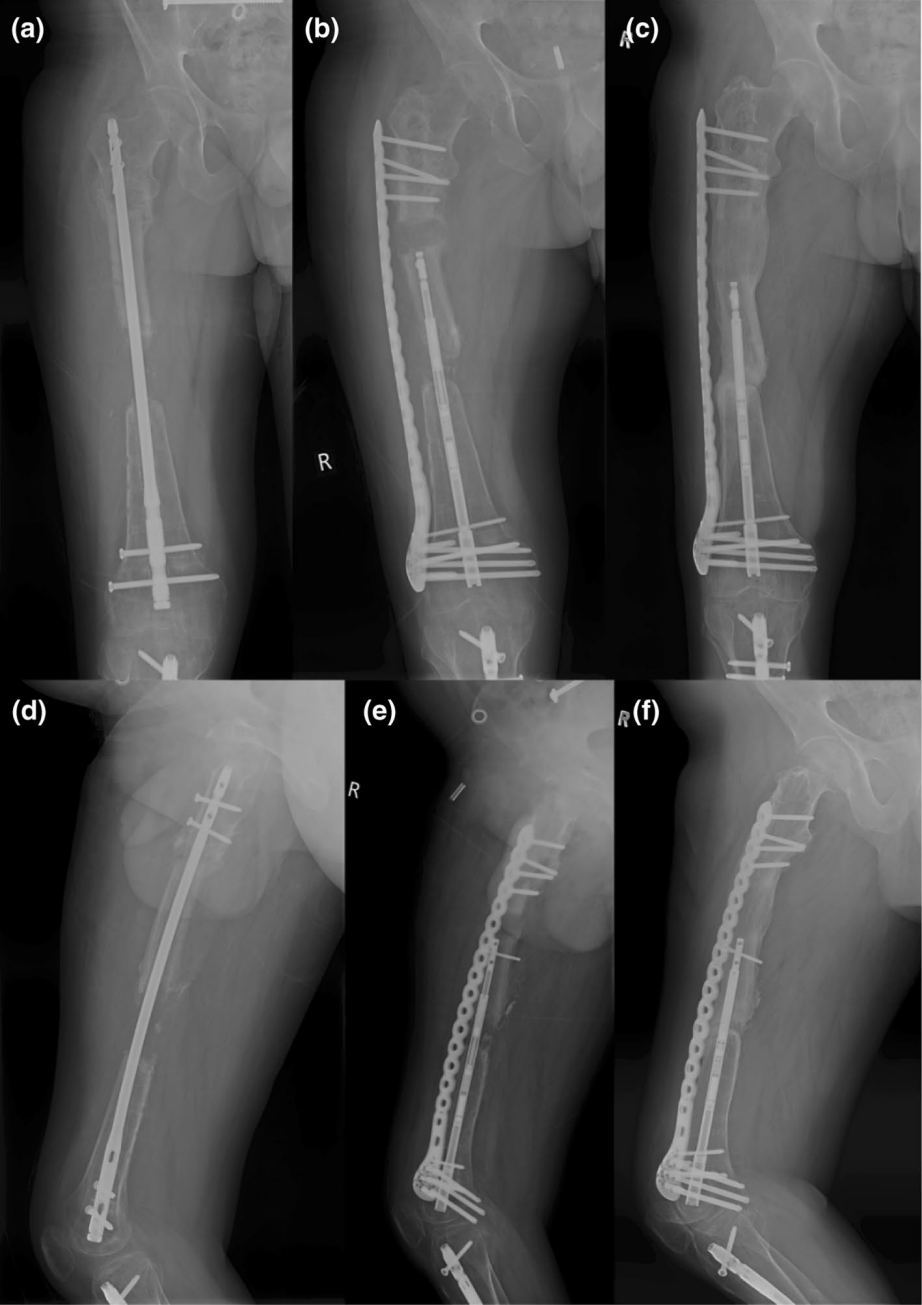
AP (**a**, **b**, **c**) and lateral (**d**, **e**, **f**) radiographs of Patient 1 showing the defect at the start, during the retrograde bone transport and at the final stage. Patient 1 had a previously unsuccessful bone transport by MRS Ex-Fix before using magnetic lengthening nails

**Fig. 2:**
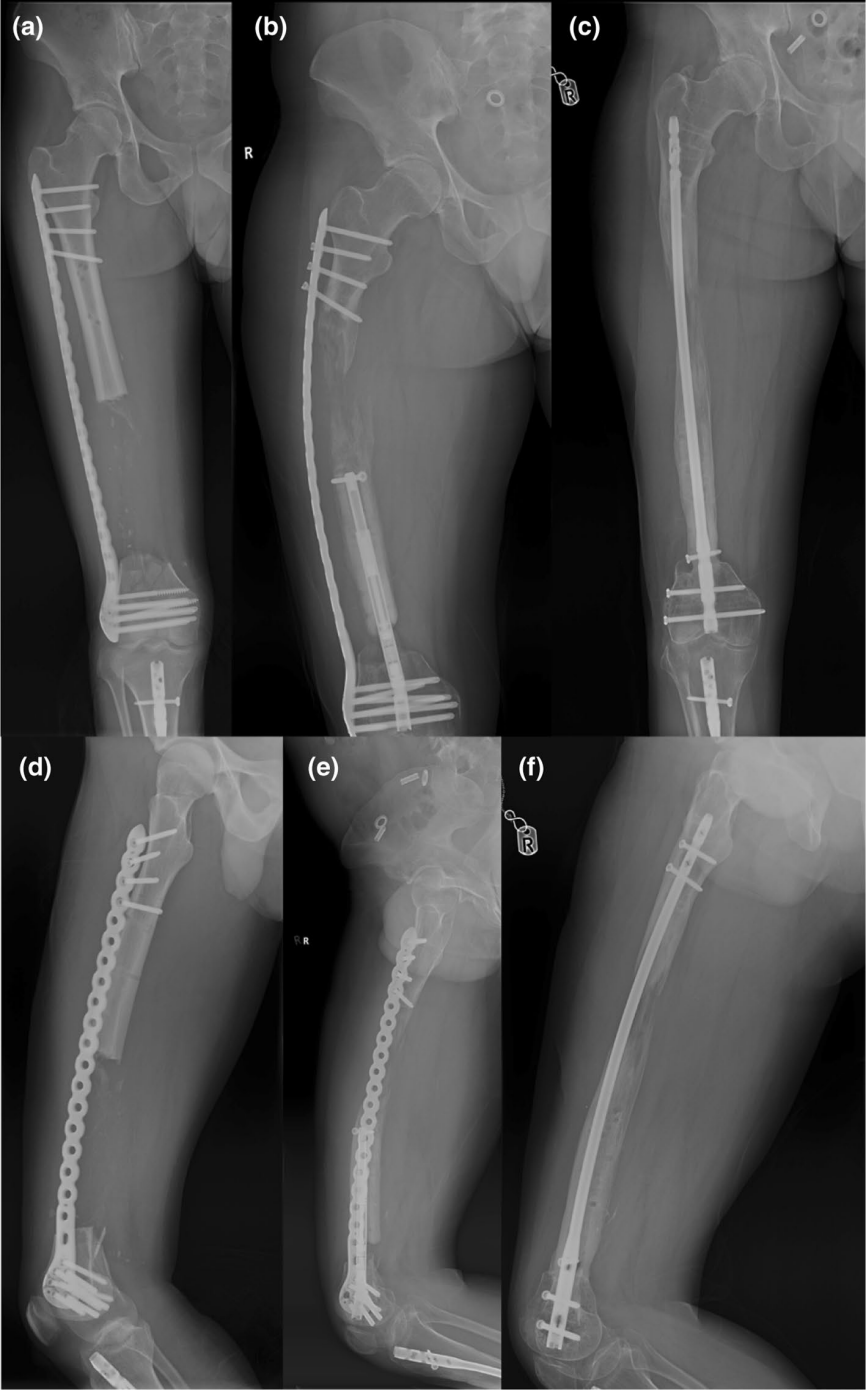
AP (**a**, **b**, **c**) and lateral (**d**, **e**, **f**) radiographs of Patient 3 showing the defect at the start, during the retrograde bone transport and at the final stage. This demonstrates plate bending, as seen in two of our patients

**Fig. 3:**
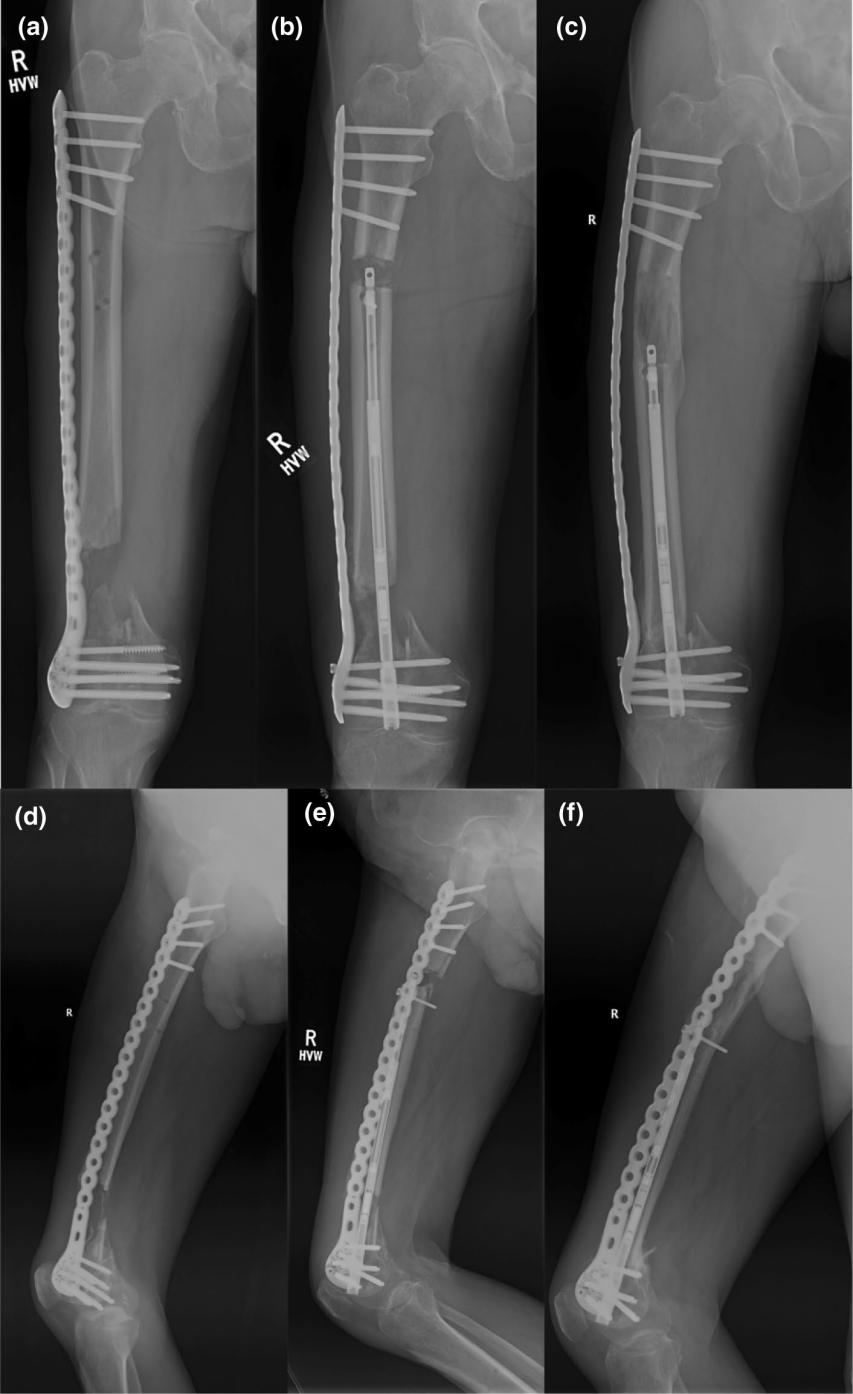
AP (**a**, **b**, **c**) and lateral (**d**, **e**, **f**) radiographs of Patient 4 showing the defect at the start, during the retrograde bone transport and at the final stage

X-ray radiographs were used to assess progress before, during and after bone transport (Figs. 1, 2, 3).

## Discussion

Bone transport with magnetic nails is a relatively novel technique in the armamentarium for long bone defects. Currently, few studies exist in the literature, mainly as case series or case reports, which aim to describe the procedure and characterise the outcomes of interest. The picture of patient experience and procedure outcome remains unclear. Consolidation of the outcome picture will strengthen its reliability as a tool for surgeons and identify any possible association such that its shortcomings may be improved.

Firstly, the treatment technique has classically been dictated by the defect size: either bone transport or acute shortening and re-lengthening. A defect size of 4–5 cm in the tibia and 6–7 cm in the femur are conventionally held as the limits for choosing acute shortening, [14] while bone transport is reserved for larger defects. Whilst we accept these criteria, we only acutely shorten if there is no other option. Our selection partially overlaps with what would have traditionally been managed with shortening. Similarly, Olesen et al. [12] treaded on the same boundary with a mean femoral defect of 9.3 cm (range 7–11.5) and a mean tibial defect of 8.9 cm (range 4.8–15). Wright et al. [13] reported a mean femoral defect size of 10.3 cm (range 7–16). Barinaga et al. [11] reported on a traumatic tibial fracture of 3.0 cm when PABST was indicated, after previous IM nail failure when the initial defect was 4.2 cm. The authors acknowledged that acute shortening and distraction would have normally been indicated for his defect size. Together, this highlights the need to elucidate when bone transport with magnetic nails should be employed over acute shortening, and, if any, a lower limit of defect size. Our study echoes the obscurity in the suitability of this technique for smaller defect sizes, often overridden by expert opinion or patient choice. This is an area that warrants assessment in a future study. It is essential to clearly demonstrate the absence of major complications and a faster return to function in this technique over acute shortening.

Secondly, while bone transport with magnetic nails has been used in both femoral and tibial bone losses, the scarcity of cases have warranted little differentiation between the two in early reports. Konofaos et al. [6] presented a mixed report with femoral and tibial cases, while Barinaga et al. [11] exclusively reported on three femoral cases. Olesen et al. [12] reported on both, but separated the two in their analysis and drew very different outcome pictures. They reported on nine patients, of these only five are femoral cases. It appears critical to differentiate the two locations for they present differently, and thus our study maintained homogeneity with only femoral cases.

The causes of bone loss should also be considered, which are broadly from trauma, infection or post-tumour resection. Olesen et al. [12] reported five femoral defects, of which three were from trauma, one from osteosarcoma and one from thyroid metastasis. Konofaos et al. [6] reported five post-tumour resection while Wright et al. [13] and Barinaga et al. [11] reported exclusively traumatic bone loss. Any differences have not been clearly detected and stated, but it is conceivable that a bone tumour pathology would result in longer follow-up due to the additional use of adjuvants. While defect lengths can be the same, post-traumatic or post-infection defects behave differently compared to tumour-related defects mainly from the infection perspective and complications. To illustrate, Olesen et al. [12] reported longer follow-up in their non-traumatic cases (average 34.5 mo) compared to traumatic cases (average 19 mo). Konofaos et al.’s [6] cases were all on chemotherapy, although their reported follow-up did not reflect this. Our series (average 21.3 mo) included only traumatic bone loss to maintain homogeneity of our sample.

In this study, we assess the outcomes of our intervention to highlight different aspects of its utility, which can be broadly categorised into functional recovery, radiological evidence, follow-up and complications. There is clearly variation in the details that are being reported. We attempt to establish essential outcome reporting, in order to accurately characterise the effect of this intervention.

### Functional outcome

One major aspect in the assessment of functional recovery is consolidation index. Our mean index of 0.8 mo/cm closely matches to that of 0.9 mo/cm reported by Olesen et al. [12] in the femur. However, a direct comparison may be rendered invalid as we adopted different key term definitions. Their consolidation index was defined as ‘the time from corticotomy to radiographic consolidation… divided by the transport distance’ [12]. They also reported a mean tibial consolidation index of 1.26 mo/cm [12], which is similar to that of 56.9 days/cm reported by Barinaga et al. [11] with the Accordion technique, further highlighting the need to separate the bones in reporting. However, Barinaga et al. [11] did not define their consolidation index, which renders any direct comparison obscure. In addition, consolidation indices were not consistently reported in other studies. Wright et al. [13] deliberately did not report on consolidation index because it was deemed redundant by their method. Besides PABST, they introduced additional interventions such as medial plating and immediate physiotherapy and weight-bearing during transport. Rather than a straight assessment of PABST, their study claimed to act as a proof of concept for these additional interventions to allow earlier return to function, thus negating the need for reporting the consolidation time.

Our definition of consolidation index allowed us to bypass the implant’s limitations, by starting at the end of bone distraction rather than the time from corticotomy. The magnetic implant is limited by the nail length and device failure. While repeated use with the PRECICE nail left in situ is an established practice in the literature, recharges are sometimes required to achieve full lengthening. The stroke size of nail was insufficient in large bone defects such that one of our cases needed three recharges. Wright et al. [13] similarly reported two cycles of lengthening with the same nail in three patients. Another limitation is device failure. This is commonly caused by failed communication between the ERC and the magnet within the nail, which is critical for nail lengthening post-operatively. An increased distance between the ERC and magnet in obese patients due to greater proximal soft tissue depth may limit the use of an anterograde approach, thus a retrograde approach is preferred. In our series, we encountered one case of device failure, which was later found to be due to nail jamming as a result of three point loading of the nail during the shortening phase. This time difference may be subtle in uneventful surgeries, but have the capacity to cause significant variations in small samples where implant limitations may confound the results. It is generally accepted that consolidation index is an important aspect of outcome, and any meaningful comparison between studies requires a consistent key term definition.

Another key aspect of assessing functional outcome is time to full weight-bearing. Our average time to full weight bearing is 18.7 mo (range 9.1–30.6), while Olesen et al. [12] reported 5 mo (range 4–6). They allowed 10–20 kg of weight-bearing during transport, and then as tolerated after docking, but recommended waiting until full consolidation of regenerate before full weight-bearing. Our study employed similar advice, but the large difference in time to weight-bearing may be due to a number of factors. Three factors are discussed as follows. Firstly, this can be partially attributed to key term definition again: while time to full weight-bearing is often taken to be from the injury date to full weight-bearing, large variations may arise if PABST was not the primary indication but other techniques were trialled and failed first. Barinaga et al. [11] reported a time to weight-bearing of POD 156, which was similar to Olesen et al.’s. Although not specified, it is a reasonably guess that Olesen et al. may have adopted the same approach.

Secondly, patient-specific factors may have contributed to the large time difference as three of our cases did not weight-bear during transport due to slow regenerate. Slow regeneration can be potentially attributed to the diaphyseal location of corticotomy [11] or the transport rate being too fast. We allowed 0.25 mm 4 times daily. There is a slight variation in the transport regimen in the literature. Barinaga et al. [11] opted for 0.25 mm 3 times daily which was followed by the Accordion technique to compensate for slow regeneration. Wright et al. [13] followed 0.33 mm 3 times daily which was complemented by double-plating and early physiotherapy and full weight-bearing. Olesen et al. [12] adopted a mix of 0.25–0.33 mm 3–4 times daily, dependent on patient factors (e.g. smoker or elderly), and moderated from reviewing bone formation during follow-ups. We permitted a similar flexibility in our regimen by allowing Patient 3 to reduce rate to 0.5 mm/day to avoid plate breaking and then increased the rate to 1.6 mm/day for 2 weeks after replacing his nail. However, the nail continued to jam so we switched to monorail ex-fix. Other cases in our series tolerated our standard rate and had no changes during the course of bone transport. Our transport regimen is very similar to that of Olesen et al.’s, therefore the cause of slow regenerate may be attributed to elsewhere.

Finally, delayed virtual follow-up assessments during the COVID-19 pandemic in two of our cases may have had a minor contribution to a longer time to full weight-bearing.

The last aspect of functional recovery assessment is joint ROM, which was assessed in follow-up clinics. Our average knee ROM is 8–107 and Olesen et al. [12] reported 0–150. Their post-intervention range appears to be greater than the normative reference values reported by McKay et al. [21], which are 1–136 (males) and 2–137 (females). Wright et al. [13] reported two of three cases achieving full knee ROM and full weight bearing during the bone transport phase, despite still consolidating at the docking site at the time of their study. Their intervention may be too different from ours in order to make a valid comparison, but they serve to illustrate the possibility of a faster functional recovery with a more aggressive approach. Konofaos et al. [6] only reported a simple subjective grading of lower limb function (i.e. ‘Excellent/Good’ with no specified ranges) without joint ROM or time to full weight bearing, which was inadequate for comparison. In addition, we find that typically only knee ROM is reported in the literature, while we think it may be worthwhile to also assess hip ROM too in femoral cases.

Besides the aforementioned outcomes, an addition of PROMs would provide a valuable aspect of lower limb functional recovery. Although PROMs were commonly not reported, we attempted to report this post-intervention, but we were unable to obtain pre-injury data for intra-study comparison and thus used population norms instead. We performed a one-tailed one-sample t-test and our series found a statistically insignificant mean EQ-5D index (*M* = 0.536, SD = 0.324) compared to that found in the UK population as a whole, *t*(3) = 1.98, *p* = 0.071. Similarly, we found no significant mean EQ-5D VAS (*M* = 51.3, SD = 31.2) compared to the population norm, *t*(3) = 2.02, *p* = 0.068. When interpreted together with the OKS and SF-36 outcomes, which show largely reduced functions across all health dimensions, the resulting clinical picture appears conflicting. Pre-injury data and a larger sample size are needed to determine if the intervention decreases disease burden.

### Radiological outcome

Radiological outcome is sometimes reported as time to union, whereby radiological assessment is used. Our mean time to union was 18.1mo (range 7.3–30.6), while Olesen et al. [12] reported 8.5 mo (range 5–12) in the femur and 7.5 mo (range 6–9) in the tibia. Barinaga et al. [11] reported 18 mo (defined as after PRECICE 2 IM nail placement to radiographic evidence) for their single tibia case. This large variation can be accounted for by time to union definitions, frequency of imaging and patient factors.

### Follow-up

Follow-up as an outcome was unexpectedly under-reported. Our mean follow-up is 21.3 mo (range 6.8–33.0). Olesen et al. [12] reported an average follow-up of 25 mo (range 18–36) for femoral cases and 10.5 mo (range 4–13) for tibial cases, although this outcome measure was not consistently reported by other authors. Konofaos et al. [6] reported an average follow-up of 11 mo (range 6–17), but proposed that more data are required to conclude the technique’s long-term structural integrity. In their case, it may be that follow-up had not been carried out over a sufficient time period. We believe that follow-up is needed to determine the long-term reliability of this technique.

### Complications

General complications associated with bone transport are heterotrophic ossifications, soft tissue contracture, superficial infection, delayed union of docking sites, reduced joint movement, and malalignment resulting in varus deformities. In our series, patients reported pain from heterotrophic ossification, slow regenerate, reduced knee ROM, mild varus deformity, two cases of plate bending, and one case of nail failure. We had one case of minor varus deformity which was left uncorrected. Significant malalignment that develops post-operatively could be corrected with a secondary trauma nail or by relocking the plate to the bone [12]. Our reported set of complications are very similar to those reported in the literature. Konofaos et al.’s [6] reporting of complications was limited to either infection or hardware failure. Barinaga et al. [11] reported slow regenerate and non-union while Wright et al. [13] reported knee stiffness, tibial fracture on manipulation. Olesen et al. [12] reported heterotrophic ossification, contracture, delayed union, superficial infection and mild varus deformity.

As illustrated, significant inter-study differences exist in the current literature for the intervention bone transport with magnetic nails in LBDs, rendering most comparisons invalid. We have demonstrated that it is difficult to extract useful conclusions about the characteristics and application of this technique. However, the current studies provide some scope for such technique as a management option for large femoral BDs in different clinical scenarios, whilst identifying potential complications.

### Limitations

The strengths of our study are the homogeneity of patients and fracture type, PROMs and consistency of the surgical technique, while our main shortcomings are small sample size, retrospective design and lack of baseline assessment in PROMs. Most case reports provided a detailed description of the surgical technique, pre- and post-operative care. We did not provide a description here because we have taken a standard approach.

### Future directions

A review of the current literature along with our study elucidated limitations in our current understanding of the technique bone transport with magnetic nails. A comprehensive reporting of outcomes is essential for valid comparisons between case series, providing key term definitions. Our study reported on exclusively traumatic femoral defects. Maintaining defect homogeneity by location and pathology in participant selection can reveal a different outcome picture and may uncover valuable insights into the suitability of this technique in various clinical scenarios. Finally, a more rigorous establishment of the technique’s indication with regards to defect size is needed, and particularly its indications over acute shortening and re-lengthening for smaller defect sizes. We propose a standard reporting template (Table 3) for future bone transport studies to enable easier meta-analysis in the future.

**Table 3:** A proposed standard reporting template for future bone transport studies

Part	Item #	Checklist item
A—Patient characteristics	1	The report contains basic details of age, sex, co-morbidities and other treatments/features which may influence follow-up
	2	The report contains information on defect pathology (e.g. traumatic, infection, tumour), defect size (primary and/or secondary if other techniques had been trialled first, distraction distance if incomplete transport and length difference), defect location (laterality and bone(s) involved)
B—Method	1	The report provides adequate description of technique employed, surgical modality, implants/devices used and whether carried out by same surgeon. If the key technique was adapted or if other techniques delivered, then describe why, when and how
	2	The report outlines the clinical management of patient (e.g. pre-operative and post-operative care)
	3	Any significant time delays that may influence the measured time-dependent outcome
C—Functional outcome	1	Consolidation time/index*, time to full weight-bearing*, adjacent joint ROMs, PROMs (e.g. EQ-5D-5L, SF-36) for both pre- and post-intervention
D—Radiological outcome	1	Time to union*
E—Other characteristics	1	Follow-up*, complications, additional notes

*Consolidation time/index, time to full-weight bearing, time to union and follow-up are all time-dependent outcomes, hence a clear definition must be provided

## Conclusion

In conclusion, bone transport with magnetic nails for long bone defects remains a relatively unchartered territory, principally backed by expert opinion and anecdotal evidence. We have contributed one perspective of its use in traumatic femoral defects with a small sample of five, and have attempted to identify key outcome measures that would assist in building a meaningful picture of patient experience and procedure outcome.
